# Relación de la calidad y la duración del sueño en población colombiana con hipertensión arterial

**DOI:** 10.7705/biomedica.7012

**Published:** 2024-05-31

**Authors:** Ludwing Ricardo Marín, Lina María Vera, Boris Eduardo Vesga, Mabelyn Solany Marín

**Affiliations:** 1 Grupo de Investigación GERMINA, Hospital Universitario de Santander, Universidad Industrial de Santander, Bucaramanga, Colombia Universidad Industrial de Santander Universidad Industrial de Santander Bucaramanga Colombia; 2 Departamento de Salud Pública, Universidad Industrial de Santander, Bucaramanga, Colombia Universidad Industrial de Santander Universidad Industrial de Santander Bucaramanga Colombia; 3 Grupo de Investigación GERMINA, Instituto del Corazón de Bucaramanga, Departamento de Medicina Interna, Universidad Industrial de Santander, Bucaramanga, Colombia Universidad Industrial de Santander Universidad Industrial de Santander Bucaramanga Colombia; 4 Departamento de Medicina Interna, Universidad Industrial de Santander, Bucaramanga, Colombia Universidad Industrial de Santander Universidad Industrial de Santander Bucaramanga Colombia

**Keywords:** hipertensión, presión arterial, higiene del sueño, trastornos del sueño- vigilia, medicina del sueño., Hypertension, arterial pressure, sleep hygiene, sleep wake disorders, sleep medicine specialty.

## Abstract

**Introducción.:**

Las alteraciones en la calidad y la duración del sueño son factores de riesgo para el desarrollo de hipertensión arterial sistémica en los países orientales. Sin embargo, hay pocos estudios de los países de Latinoamérica para investigar esta asociación. Objetivo. Analizar la asociación entre la calidad y la duración del sueño, y la incidencia de hipertensión arterial sistémica en población colombiana.

**Materiales y métodos.:**

Se llevó a cabo un estudio observacional, longitudinal, prospectivo y analítico, anidado en la cohorte de base poblacional INEFAC, desarrollado con participantes mayores de 18 años de Bucaramanga (Colombia). El sueño se evaluó mediante el índice de calidad del sueño de Pittsburgh y, su duración, mediante preguntas estandarizadas. Se realizó un análisis multivariado con modelos de regresión logística ajustados por las posibles variables de confusión.

**Resultados.:**

Se incluyeron 1.306 participantes no hipertensos con edad media de 40 ± 12 años. El 92,8 % de la población presentaba algún problema del sueño, el 45,15 % dormía 6 horas o menos y el 28,6 % dormía 8 horas o más. El análisis multivariado mostró un mayor riesgo de hipertensión en los participantes con diabetes (OR = 5,27) (IC_95%_: 2,27-12,26), obesidad (OR = 2,81) (IC_95%_: 1,11-7,13), tabaquismo activo (OR = 2,02) (IC_95%_: 1,01-4,04) y mayor estrato socioeconómico (OR = 4,94) (IC_95%_: 1,59-15,38 para estrato 4), pero no se encontró un mayor riesgo en los participantes con mala calidad o poca duración del sueño.

**Conclusiones.:**

No se demostró asociación alguna entre la duración o la calidad del sueño y la incidencia de hipertensión arterial sistémica en población colombiana. Se requieren más estudios en esta población para llegar a conclusiones definitivas.

## Abstract (en)

**Introduction.:**

Alterations in the quality and duration of sleep are risk factors for the development of arterial hypertension in Eastern countries. However, in Latin America there are few studies researching this association.

**Objective.:**

To analyze the association between the quality and duration of sleep and the rate of arterial hypertension in a Colombian population.

**Materials and methods.:**

An observational, longitudinal, prospective and analytical study nested in the INEFAC population-based cohort, was conducted with participants over 18 years of age from Bucaramanga (Colombia). Sleep quality was assessed using the Pittsburgh Sleep Quality Index. Sleep duration was assessed using standardized questions. Multivariate analysis was performed with logistic regression models adjusted for possible confounding variables.

**Results.:**

A total of 1,306 non-hypertensive participants with a mean age of 40 ± 12 years were included. In this population, 92.8% had one or more sleep issues. 45.15% slept 6 hours or less and 28.6% slept 8 hours or more. Multivariate analysis showed a higher risk of hypertension in participants with diabetes (OR = 5.27; 95% CI: 2.27-12.26), obesity (OR = 2.81; 95% CI: 1.11-7.13), active smoking (OR = 2.02; 95% CI: 1.01-4.04) and higher socioeconomic level (OR = 4.94; 95% CI: 1.59-15.38 for level 4), but no higher risk was found in participants with poor sleep quality or short sleep duration.

**Conclusions.:**

No association was found between the duration or quality of sleep and the rate of arterial hypertension in the Colombian population. More studies are required in this population to reach definitive conclusions.

Las enfermedades cardiovasculares ocupan el primer lugar en la morbimortalidad causada por enfermedades no transmisibles ^1^. La hipertensión arterial sistémica es el principal factor de riesgo para el desarrollo de enfermedades cardiovasculares a nivel global ^2^. Se estima que en el mundo hay 1.280 millones de adultos de 30 a 79 años con hipertensión arterial, y que la mayoría de ellos vive en los países de ingresos bajos y medios ^3^.

En Colombia, para el año 2021, la hipertensión arterial fue la sexta causa de muerte en el país, con una tasa de mortalidad de 312,74 por 100.000 habitantes, más del doble de la tasa registrada para el año 2018 ^4^. La detección temprana de la hipertensión arterial junto con un tratamiento apropiado contribuyen a mejorar la calidad de vida, y disminuir las complicaciones y los costos; por esta razón, es importante conocer los diferentes factores de riesgo que intervienen en el desarrollo de la enfermedad.

La industrialización, la globalización y el entorno muy productivo que nos hemos autoimpuesto y en el que nos desenvolvemos en la actualidad, han producido cambios significativos en el estilo de vida de los seres humanos, incluyendo cambios en los hábitos de sueño ^5^. El trabajo por turnos, la duración corta del sueño y el síndrome de apnea se han descrito como factores de riesgo para hipertensión arterial ^6^. Cada vez es más frecuente el sueño insuficiente y de mala calidad, lo cual es atribuible al insomnio y a su restricción voluntaria, principalmente, por un aumento en la carga laboral y las exigencias de la vida moderna en general ^7^^-^^10^.

En Colombia, la prevalencia de los trastornos del sueño es del 27 %, por lo que se trata de un problema de salud pública y, entre ellos, resaltan la apnea y el síndrome apnea, con una prevalencia global de alto riesgo del 19 % (IC_95%_: 17,3; 20,8 %) y del 26,9 % (IC_95%_: 24,9; 29,0 %), respectivamente ^11^^,^^12^. La relación entre este síndrome y los eventos cardiovasculares ya ha sido establecida previamente en la literatura científica. En los individuos con síndrome de apnea, la prevalencia de enfermedad cardiovascular es dos a tres veces mayor que en la población general ^11^.

También, se ha presentado un aumento de la prevalencia en la hipertensión arterial en los últimos años ^4^ y, aunque ya se encuentran establecidos algunos factores de riesgo para su desarrollo, recientemente se ha mostrado un interés por nuevos factores que parecen predisponer a una mayor probabilidad de padecerla, como son las alteraciones del sueño ^13^^,^^14^.

En los países orientales, existe evidencia a favor de una asociación entre la mala calidad y la poca duración del sueño, con una mayor prevalencia de hipertensión arterial ^13^^,^^14^. Sin embargo, en Latinoamérica hay pocos estudios que investiguen esta asociación y en ninguno de ellos se utilizan escalas validadas, como el índice de calidad del sueño de Pittsburgh (PSQI), como una herramienta menos subjetiva para medir la calidad del sueño.

El objetivo de este estudio fue analizar la asociación entre la calidad y la duración del sueño, con la incidencia de hipertensión arterial en una población del nororiente colombiano.

## Materiales y métodos

Se llevó a cabo un estudio observacional, analítico y de cohorte prospectiva, anidado en la cohorte de base poblacional INEFAC (incidencia de enfermedades cardiovasculares y sus factores de riesgo) de fase I y II, realizada en Bucaramanga (Santander) entre los años 2007 y 2017.

La información de la línea de base de la cohorte INEFAC fue recolectada en los años 2000-2001, en el programa CARMEN (conjunto de acciones para la reducción multifactorial de las enfermedades crónicas no transmisibles). En el presente estudio se utilizaron los datos obtenidos del primer seguimiento de la cohorte en el año 2007, conocido como INEFAC I, y del segundo seguimiento llevado a cabo entre los años 2013 y 2017, conocido como INEFAC II. Los métodos utilizados en el estudio INEFAC han sido publicados previamente ^15^.

La muestra del programa CARMEN fue constituida por adultos mayores de 18 años y fue obtenida mediante un muestreo aleatorio por conglomerados a partir de 40 barrios (conglomerados) de estratos 2 y 3 de Bucaramanga. En cada barrio, se seleccionaron al azar alrededor de 60 a 80 casas y, finalmente, en cada casa se seleccionó un participante al azar. La población de base de la cohorte CARMEN incluyó 2.432 participantes.

En el 2007, se logró contactar a 1.626 participantes que constituyeron el primer seguimiento de la cohorte (INEFAC I), de donde se tomó la muestra para el presente estudio. En el segundo seguimiento (INEFAC II) se logró contactar 1.148 participantes y este constaba de dos etapas; la primera se llevó a cabo en el año 2013 y, la segunda, en donde se hizo seguimiento a la mayoría de la población, durante 2016 a 2017.

Para cada seguimiento, se solicitó el consentimiento informado, y se hizo una encuesta estructurada, usando medidas antropométricas y exámenes de laboratorio. Cada participante recibía un código de 6 dígitos asignado desde la encuesta basal, para salvaguardar su identidad durante el proceso de análisis de los datos. Los participantes fueron evaluados por personal de salud capacitado y entrenado.

Se elaboraron manuales de procedimientos, los investigadores recibieron capacitación y se divulgaron estos manuales para poder recolectar los datos, lo que llevó a mediciones estandarizadas.

El control de calidad de los datos se hizo mediante verificación diaria de las encuestas, las medidas físicas y los exámenes de laboratorio. Se practicaron pruebas de laboratorio por duplicado con laboratoristas enmascarados, en una muestra aleatoria del 10 %, para verificar posibles discordancias o errores en la toma o en el procesamiento de las muestras. Las medidas del examen físico también se tomaron por duplicado. Se llevó a cabo una doble digitación con el *software* Epilnfo, versión 6.04, con digitadores independientes y validación de datos.

Los criterios de inclusión y exclusión son los mismos utilizados en la cohorte original. Los criterios de inclusión fueron: haberse sometido a la medición de la presión arterial en las dos fases de seguimiento INEFAC I y II, y haber respondido el formulario de calidad del sueño; no estar embarazada; ser residente en el área urbana de la ciudad; y estar en capacidad de responder una entrevista verbal y de sostenerse en posición de bipedestación.

Los criterios de exclusión incluyeron estar trabajando en horario nocturno durante el último mes y tener diagnóstico previo o *de novo* de hipertensión arterial sistémica durante el seguimiento de 2007.

El tamaño de la muestra se calculó con base en los resultados obtenidos por Liu *et al.*^16^ con un nivel de confianza del 95 % y un poder del 80 %. Se calculó que, para encontrar una diferencia significativa se requerían, para calidad del sueño, 134 individuos para una relación entre no expuestos y expuestos de 1:1 y, 203 individuos, para una relación entre no expuestos y expuestos de 1:4. Para la duración de sueño, se requerían 494 participantes para una relación de no expuesto y expuestos de 1:1 y, 764 participantes, para una relación de no expuestos y expuestos de 1:4.

La descripción de las variables se muestra en el cuadro suplementario 1.

En el caso de la variable dependiente, incidencia de hipertensión arterial, la presión arterial se midió teniendo en cuenta los criterios recomendados por la American Heart Association. Se utilizó el monitor de presión arterial automático elite, OMRON HEM-7320. Antes de la toma de la presión arterial, se le preguntó al participante acerca de su última comida, consumo de café, chocolate o cigarrillos en las últimas 8 horas, actividad física y consumo de medicamentos previos a la cita.

La presión arterial se midió después de que el participante descansara durante cinco minutos en posición sedente; se midió el perímetro braquial para utilizar el brazalete adecuado; el brazalete se colocó sobre la arteria braquial con el brazo del participante a la altura del corazón; después de cada medición, el paciente descansaba durante dos minutos para pasar a la siguiente medición. Se tomaron en total tres mediciones y, para clasificar al paciente como hipertenso, se tuvo en cuenta el promedio de las dos últimas mediciones.

Se consideraron hipertensos aquellos participantes con una presión arterial de 140/90 mm Hg y aquellos con diagnóstico previo de hipertensión o que estuvieran tomando antihipertensivos.

Las variables independientes comprendidas, como calidad y duración del sueño, se valoraron mediante el PSQI-un cuestionario validado en Colombia-que cuenta con 19 preguntas para evaluar la autopercepción de la calidad del sueño y sus alteraciones durante el último mes y, además, y contiene cinco preguntas adicionales dirigidas al compañero de cama en caso de tenerlo.

Las 19 preguntas de autopercepción se distribuyeron en siete componentes (calidad subjetiva del sueño, latencia, duración, eficiencia habitual, trastornos, uso de medicamentos para dormir y disfunción diurna). Cada pregunta se calificó de 0 a 3 con un posible puntaje final entre 0 y 21 puntos; 0 indicaba ausencia de problemas con el sueño y, 21, dificultad grave, para todos los componentes; además, un valor mayor o igual a 5 indicaba mala calidad del sueño.

El puntaje total de la escala PSQI se clasifica de la siguiente forma: sin problemas del sueño (≤ 4 puntos), problema del sueño que merece atención médica (5-7 puntos), problema del sueño que merece atención médica y tratamiento (8-14 puntos), problema grave del sueño (15-21 puntos) ^17^.

Por otro lado, la duración del sueño como variable independiente se categorizó en una duración menor o igual a 5 horas y 6 horas o mayor o igual a 8 horas y 9 horas, categorías que se compararon con 7 horas en los análisis estadísticos. El número de horas de sueño se tomó del reporte del participante del número de horas que creía que dormía cada noche.

Las variables sociodemográficas y de confusión o modificadores de efecto, se describen en el cuadro suplementario 1.

Se exploraron valores extremos, valores perdidos, valores máximos y mínimos, escalas de medición, doble digitación y adecuada codificación de los datos. Para el análisis estadístico, se utilizó el *software* Stata 14.0™. Se hizo un análisis descriptivo para obtener la frecuencia absoluta de las variables de interés. Las variables categóricas se describieron mediante su frecuencia absoluta y su respectiva proporción, y las variables continuas, con medidas de tendencia central y de dispersión.

Se presentan los datos diferenciados por sexo y, para determinar si había diferencias por sexo, se aplicó la prueba de ji al cuadrado para las variables categóricas y la prueba paramétrica t de Student para las variables cuantitativas continuas. Posteriormente, se hizo un análisis bivariado para evaluar la asociación entre la variable dependiente (incidencia de hipertensión arterial) y las variables independientes principales (alteración en la calidad del sueño medida mediante el PSQI y la duración del sueño). Se estimó la asociación cruda con las demás variables independientes de interés (posiblemente de confusión).

Para evaluar la asociación entre variables cuantitativas y variables cualitativas dicotómicas con distribución normal, se utilizó la prueba paramétrica t de Student y, en caso de distribución no normal, la prueba no paramétrica de U de Mann-Whitney. Para evaluar la asociación entre incidencia de hipertensión arterial y las demás variables cualitativas categóricas independientes, se usó la prueba de ji al cuadrado. Las asociaciones se consideraron estadísticamente significativas con un alfa menor de 0,05.

Para el análisis multivariado, con el fin de evaluar la asociación de alteración en la calidad y duración del sueño, con la incidencia de hipertensión arterial, se usó un modelo de regresión logística ajustado por las variables identificadas previamente en la literatura científica como de confusión o modificadoras del efecto y aquellas que en el análisis bivariado mostraron una p < 0,20.

Se realizó un análisis de pérdidas para determinar si las características de los participantes no evaluados (perdidos) en el segundo seguimiento de la cohorte diferían de las características de los participantes que se lograron contactar nuevamente. Para esto, se construyó una variable para predecir la participación en el segundo seguimiento que se llamó “participación (sí o no)”, y se realizaron modelos bivariados con las variables independientes.

Posteriormente, se construyó un modelo de regresión para observar la probabilidad de participación, teniendo en cuenta las variables que podrían estar asociadas con el resultado. Se predijo la probabilidad de participar o no en el estudio y, a cada sujeto, se le asignó una probabilidad de estar expuesto o no, en función de los factores de confusión. Finalmente, utilizando el método de propensión, se crearon los pesos muéstrales con el inverso de la probabilidad y, con este, se ajustó el modelo final.

### Consideraciones éticas

Este estudio fue avalado por el Comité de Ética de la Universidad Industrial de Santander. Se contemplaron los principios de beneficencia y no maleficencia. Para este análisis, se recurrió a la información recolectada con el consentimiento de los participantes de la cohorte INEFAC.

## Resultados

**Figura 1.**
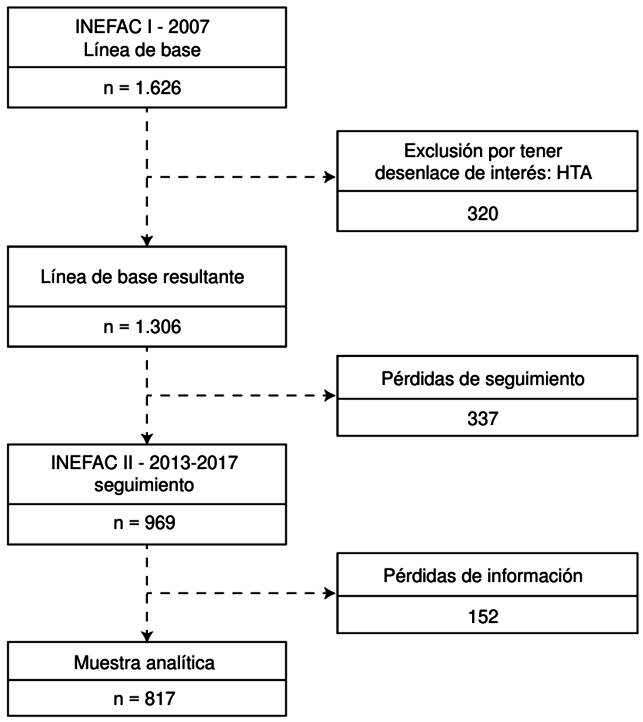
Flujograma de la población en estudio

De los 1.626 participantes de INEFAC I, se excluyeron 320 que eran hipertensos, pues ya presentaban el evento de interés, por lo cual los seleccionados para el presente estudio fueron en total 1.306 (figura 1). La edad media de los participantes fue de 40 años (DE ± 12 años) (IC_95%_: 39,05-40,38). La proporción de mujeres fue del 68,7 %. En el cuadro 1 se muestran las características generales y sociodemográficas por sexo de la población en la línea de base.

**Cuadro 1.** Características generales y sociodemográficas por sexo de la población en la línea de base 2007

Variable		***Total (N*=1306V**		Hombres (n=409)		Mujeres (n=8 97)		p
		n	%	n	%	n	%	
Sociodemográficas								
Estrato								0,47^a^
	0-1	102	7,82	33	8,11	69	7,69	
	2	638	48,93	203	49,88	435	48,49	
	3	519	39,8	153	37,59	366	40,8	
	4	45	3,45	18	4,42	27	3,01	
Raza								< 0,01^a^
	Blanca	425	32,59	108	26,54	317	35,34	
	Mestiza	835	64,03	288	70,76	547	60,98	
	Negra	10	0,77	3	0,74	7	0,78	
	No responde	34	2,61	8	1,97	26	2,9	
Estado civil								< 0,01^a^
	Soltero	420	32,21	139	34,15	281	31,33	
	Casado	415	31,83	143	35,14	272	30,32	
	Viudo	51	3,91	1	0,25	50	5,57	
	Divorciado o separado	111	8,51	25	6,14	86	9,59	
	Unión libre	307	23,54	99	24,32	208	23,19	
Escolaridad								0,46^a^
	Ninguno	33	2,53	7	1,71	26	2,9	
	Primaria	439	33,61	130	31,78	309	34,45	
	Secundaria	676	51,76	219	53,55	457	50,95	
	Técnico o tecnólogo	61	4,67	18	4,4	43	4,79	
	Superior	97	7,43	35	8,56	62	6,91	
Ocupación								< 0,01^a^
	Comerciante	287	22,09	136	33,58	151	16,89	
	Profesional o administrador	109	8,39	29	7,16	80	8,95	
	Trabajos generales	322	24,79	137	33,83	185	20,69	
	Hogar	137	10,55	42	10,37	95	10,63	
	Otros	444	34,18	61	15,06	383	42,84	
Seguridad social								< 0,001^a^
	Beneficiario	294	22,6	55	13,55	239	26,7	
	Cotizante	384	29,52	152	37,44	232	25,92	
	Ninguno	623	47,89	199	49,01	424	47,37	
Comportamentales								
Tabaquismo								<0,01^a^
	No fumador	845	64,8	179	43,93	666	74,25	
	Exfumador	263	20,17	119	29,24	144	16,05	
	Fumador actual	196	15,03	109	26,78	87	9,7	
	Consumo alcohol							< 0,01^a^
	No consumidor	373	28,65	62	15,27	311	34,71	
	Consumidor	929	71,35	344	84,73	585	65,29	
Actividad física								0,05^a^
	< 600 METs/min/sem	943	81,57	285	78,08	658	83,19	
	601-1500 METs/min/sem	198	17,13	72	19,73	126	15,93	
	>1500 METs/min/sem	15	1,3	8	2,19	7	0,88	
Clínicas								
Calidad del sueño								<0,01^a^
	Sin problemas	93	7,2	37	9,23	56	6,29	
	Merece atención médica	520	40,25	183	45,64	337	37,82	
	Atención médica o tratamiento	665	51,47	176	43,89	489	54,88	
	Problema grave del sueño	14	1,08	5	1,25	9	1,01	
Horas de sueño								0,17^a^
	7 (referencia)	341	26,27	112	27,93	229	25,53	
	<5	258	19,88	68	16,96	190	21,18	
	6	328	25,27	93	23,19	235	26,2	
	8	289	22,27	100	24,94	189	21,07	
	>9	82	6,32	28	6,98	54	6,02	
Diabetes mellitus								0,90^a^
	No	1215	95,82	380	95,72	835	95,87	
	Sí	53	4,18	17	4,28	36	4,13	
Síntomas depresivos								< 0,01^a^
	Ausencia	853	65,41	301	73,96	552	61,54	
	Presencia	451	34,59	106	26,04	345	38,46	
Índice de masa corporal.								< 0,01^a^
	Bajo peso	28	2,23	10	2,54	18	2,1	
	Normal	564	45,01	205	52,03	359	41,79	
	Sobrepeso	457	36,47	142	36,04	315	36,67	
	Obesidad	204	16,28	37	9,39	167	19,44	
	Índice cintura-cadera*	0,8	±0,07	0,86	±0,06	0,77	±0,06	<0,01^b^
	Índice cintura-talla*	0,49	±0,07	0,49	±0,06	0,49	±0,07	0,97^b^

* Los datos corresponden a la media del índice cintura-cadera y cintura-talla con su respectiva desviación estándar.

^+^ Este dato corresponde al número total de participantes en la línea de base; sin embargo, el número de observaciones para cada variable no siempre suma este valor por pérdida de información de algunos participantes. Los porcentajes se calculan de acuerdo con el número de observaciones de cada variable, por lo cual siempre sumará 100 %.

^a^ ji al cuadrado

^b^ t de Student

Se encontró que el 40,25 % del total de participantes tenía una alteración del sueño que merecía atención médica, el 51,47 % tenía una alteración del sueño que merecía atención médica y tratamiento, y solo el 1,08 % tenía un problema grave del sueño. El 45,15 % de los participantes dormía seis horas o menos y el 28,59 % dormía ocho horas o más.

De los 1.306 participantes no hipertensos, se logró contactar en el segundo seguimiento (INEFACII) a 969, de los cuales 154 desarrollaron hipertensión arterial *de novo*. La incidencia de hipertensión arterial en el seguimiento a 10 años fue de 11,8 % y el puntaje de la escala PSQI en el que fue mayor, corresponde a “un problema del sueño que merece atención médica y tratamiento” con una incidencia de de la enfermedad del 9,96 %; seguido de “merece atención medica” con una incidencia de hipertensión del 7,69 %. Solo un participante con problemas graves del sueño desarrolló hipertensión arterial; sin embargo, solo hay 14 participantes en esta categoría en la línea de base, lo cual nos da una incidencia de 7,14 %. La menor incidencia se observó en los participantes sin problemas del sueño, quienes tenían una incidencia del 6,45 %.

Para el análisis bivariado, se logró obtener la información completa de 817 participantes, de los cuales 113 habían desarrollado hipertensión arterial *de novo* y 704 eran no hipertensos. Las variables que mostraron una asociación significativa con la hipertensión arterial en el análisis bivariado, fueron estrato socioeconómico, escolaridad, ocupación, seguridad social, consumo de alcohol, perturbaciones del sueño, diabetes mellitus, índice de masa corporal, índice cintura-talla e índice cintura-cadera.

**Figura 2.**
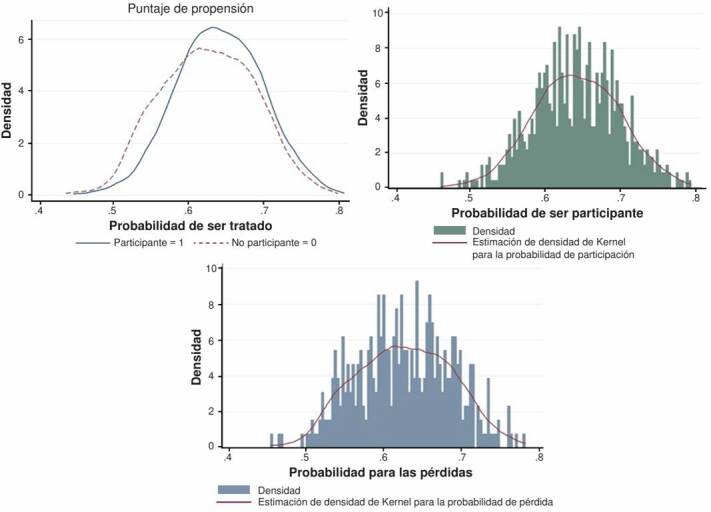
Probabilidad de participación de acuerdo con los factores de confusión

Durante el seguimiento, se observó un total de 37,4 % de pérdidas de los participantes; teniendo en cuenta que, en la mayoría de los estudios de cohorte, las pérdidas durante el seguimiento son inevitables, se hizo un análisis de pérdidas. Las características de los participantes según la probabilidad de participación se muestran en el cuadro 2. No se observó una diferencia significativa en ninguna de las variables entre los individuos que se perdieron (no participantes) y los que no se perdieron (participantes). En la figura 2 se muestra la probabilidad de participar o no en el estudio, según la probabilidad de estar expuesto o no, en función de los factores de confusión. Los resultados obtenidos en el análisis de propensión y las pruebas de hipótesis muestran que las pérdidas durante el seguimiento no fueron diferenciales.

**Cuadro 2.** Distribución de las variables sociodemográficas y clínicas según la probabilidad de participación en el estudio de incidencia de enfermedades cardiovasculares y sus factores de riesgo (INEFAC II)

		Participantes (n=817)		No participantes (n=489)		p+
		%	IC_95%_	%	IC_95%_		
Sexo (mujer)		69,77	66,49-72,90	66,87	62,50-71,03	0,11
Edad*		40,47	± 11,95	38,46	± 12,44	0,12
Estrato						0,63
	0-1	6,61	5,00-8,54	9,86	7,36-12,86	
	2	50,92	47,43-54,40	45,59	41,10-50,13	
	3	39,78	36,40-43,23	39,84	35,46-44,34	
	4	2,69	1,70-4,05	4,72	3,02-7,00	
Raza						0,99
	Blanca	32,56	29,35-35,89	32,65	28,50- 37,01	
	Mestiza	64,38	60,99-67,67	63,45	59,00- 67,74	
	Negra	0,49	0,13-1,25	1,23	0,45- 2,66	
	No responde	2,57	1,60-3,90	2,67	1,43- 4,52	
Estado civil						0,93
	Soltero	29,99	26,86-33,26	35,93	31,67-40,37	
	Casado	33,78	30,54-37,14	28,54	24,57-32,78	
	Viudo	4,9	3,52-6,61	2,26	1,13-4,01	
	Divorciado o separado	8,45	6,63-10,57	8,62	6,29-11,48	
	Unión libre	22,89	20,05-25,93	24,64	20,87-28,72	
Escolaridad						0,8
	Ninguna	2,33	1,41-3,61	2,86	1,57-4,76	
	Primaria	35,74	32,45-39,13	30,06	26,03-34,34	
	Secundaria	50,18	46,70-53,67	54,4	49,86-58,88	
	Técnico 0 tecnólogo	4,9	3,52-6,61	4,29	2,68-6,49	
	Superior	6,85	5,22-8,81	8,38	6,08-11,20	
Ocupación						0,15
	Comerciante	22,36	19,54-25,38	21,65	18,06-25,59	
	Profesional 0 administrador	8,23	6,44-10,34	8,66	6,31-11,53	
	Trabajos generales	24,57	21,65-27,68	25,15	21,35-29,26	
	Ama de casa	10,69	8,65-13,02	10,31	7,75-13,37	
	Otros	34,15	30,90-37,52	34,23	30,01-38,64	
Seguridad social						0,91
	Beneficiario	22,77	19,93-25,80	22,22	18,60-26,18	
	Cotizante	29,62	26,51-32,88	29,22	25,21-33,48	
	Ninguno	47,61	44,14-51,10	48,56	44,03-53,10	
Tabaquismo						0,15
	No	63,65	60,24-66,95	66,74	62,36-70,91	
	Exfumador	20,32	17,61-23,24	19,92	16,46-23,75	
	Fumador actual	16,03	13,58-18,73	13,35	10,45-16,69	
Consumo de alcohol						0,76
	No	28,34	25,27-31,57	29,16	25,16-33,42	
	Consumidor	71,66	68,43-74,73	70,84	66,58-74,84	
Calidad del sueño**						0,61
	Sin problemas	7,53	5,81-9,57	6,64	4,59-9,24	
	Merece atención médica	39,14	35,76-42,59	42,12	37,66-46,66	
	Merece atención médica o tratamiento	52,35	48,84-55,83	50	45,44-54,56	
	Problema grave del sueño	0,99	0,43-1,94	1,24	0,46-2,69	0,73
Horas de sueño						
	<5	20,39	17,68-23,33	19,01	15,61-22,79	
	6	25,43	22,47-28,57	25	21,20-29,11	
	7	26,54	23,53-29,71	25,83	21,98-29,97	
	8	21,25	18,49-24,23	23,97	20,23-28,03	
	>9	6,39	4,81-8,29	6,2	4,22-8,73	
Diabetes mellitus						0,64
	No	95,77	94,13-97,05	95,91	93,69-97,52	
	Sí	4,23	2,95-5,87	4,09	2,48-6,31	
Síntomas depresivos						0,73
	No	64,99	61,61-68,27	66,12	61,72-70,32	
	Sí	35,01	31,73-38,39	33,88	29,68-38,28	
Índice de masa corporal						0,98
	Normal	43,09	39,62-46,61	48,36	43,69-53,05	
	Bajo peso	2,26	1,35-3,55	2,19	1,05-3,99	
	Sobrepeso	36,81	33,45-40,27	35,89	31,48-40,47	
	Obesidad	17,84	15,24-20,68	13,57	10,56-17,05	
Índice cintura-talla						0,18
	Sin obesidad abdominal	54,91	51,38-58,41	61,84	57,21-66,32	
	Obesidad abdominal	45,09	41,59-48,62	38,16	33,68-42,79	
Índice cintura-cadera						0,93
	Sin obesidad abdominal	89,04	86,66-91,13	91,01	88,00-93,47	
	Obesidad abdominal	10,96	8,87-13,34	8,99	6,53-11,99	
Actividad física METs-min						0,98
	<600	82,27	79,29-84,99	80,88	76,85-84,47	
	> 600 y < 1.500	16,2	13,59-19,10	18,2	14,68-22,16	
	>1.500	L52	076-2.71	0.92	0 ?-5-P 34	

METs-min: equivalentes metabólicos por minuto

* Media ± desviación estándar; ** Clasificación de la calidad del sueño mediante el puntaje total del índice de Pittsburgh;^+^ Modelo de regresión probit

Finalmente, se utilizó un modelo de regresión logística para evaluar la asociación de las alteraciones en la calidad y duración del sueño, con la incidencia de hipertensión arterial. En el cuadro 3 se muestran los OR (*odd ratios*) crudos (no ajustados) y los OR ajustados de las variables sociodemográficas, comportamentales y clínicas. El modelo fue ajustado por aquellas variables identificadas previamente en la literatura científica como de confusión o modificadoras del efecto y aquellas que en el análisis bivariado mostraron un valor p < 0,20.

**Cuadro 3:** Asociación de las variables independientes con el resultado primario (incidencia de hipertensión arterial) en el seguimiento a 10 años

Variable dependiente	Variable independiente	Categorías	OR no ajustado			OR ajustado +		
			OR	IC_95 %_	p	OR	IC_95%_	p
Incidencia de hipertensión arterial	Sociodemográficas							
	Sexo	Mujeres (referencia)	---	---	---	---	---	---
		Hombres	0,76	0,49-1,21	0,26	1,00	0,55-1,83	0,99
	Edad	---	1,05	1,03-1,07	<0,001	1,03	1,00-1,05	0,02
	Estrato	2-3 (Ref.)	---	---	---	---	---	---
		0-1	0,36	0,11-1,19	0,09	0,21	0,06-0,82	0,03
		4	2,89	1,15-7,26	0,02	4,94	1,59-15,38	<0,01
	Estado civil	Sin pareja (referencia)	---	---	---	---	---	---
		Con pareja	1,05	0,70-1,56	0,82	0,93	0,56-1,53	0,76
	Escolaridad	Ninguno o primaria (referencia)	---	---	---	---	---	---
		Secundaria	0,56	0,37-0,86	<0,01	0,64	0,37-1,10	0,10
		Técnico o tecnólogo	0,23	0,05-1,00	0,05	0,35	0,08-1,63	0,18
		Superior	0,74	0,33-1,66	0,47	0,99	0,30-3,29	0,98
	Ocupación	Comerciante (referencia)	---	---	---	---	---	---
		Profesional o administrativo	0,96	0,39-2,39	0,93	1,75	0,51-5,96	0,37
		Trabajos generales	0,75	0,38-1,48	0,41	0,88	0,41-1,93	0,76
		Hogar	1,57	0,75-3,29	0,23	1,31	0,55-3,10	0,54
		Otros	1,98	1,14-3,43	0,02	1,54	0,78-3,05	0,22
	Seguridad social	Ninguna (referencia)	---	---	---	---	---	---
		Cotizante	0,7	0,42-1,17	0,18	0,68	0,36-1,28	0,23
		Beneficiario	1,53	0,96-2,43	0,08	1,16	0,64-2,09	0,62
	Comportamentales							
	Tabaquismo	No fumador (referencia)	---	---	---	---	---	---
		Exfumador	1,67	1,04-2,67	0,03	1,60	0,89-2,87	0,12
		Fumador actual	1,23	0,71 -2,14	0,46	2,02	1,01-4,04	0,05
	Consumo de alcohol	No consumidor (referencia)	---	---	---	---	---	---
		Consumidor	0,58	0,38-0,87	<0,01	0,7	0,42-1,17	0,18
	Clínicas						Clínicas	
	Calidad del sueño *	Sin problemas (referencia)	---	---	---	---	---	---
		Merece atención médica	1,35	0,55-3,34	0,51	1,6	0,49-5,23	0,44
		Merece atención médica o tratamiento	1,72	0,71 -4,15	0,23	1,57	0,36-6,85	0,55
		Problema grave del sueño	1,33	0,14-12-75	0,8	0,44	0,01-13,93	0,65
	Horas de sueño	7 (referencia)	---	---	---	---	---	---
		<5	1,35	0,78-2,35	0,29	0,79	0,36-1,76	0,57
		6	0,69	0,38-1,26	0,23	0,44	0,20-1,00	0,05
		8	1,09	0,62-1,92	0,77	0,93	0,39-2,21	0,88
		≥ 9	0,8	0,31 -2,05	0,65	0,67	0,20-2,21	0,51
	Latencia del sueño**	Sin problemas (referencia)	---	---	---	---	---	---
		Alteración leve	0,93	0,55-1,55	0,77	0,88	0,43-1,80	0,73
		Alteración moderada	1,64	1,01 -2,65	0,05	1,03	0,45-2,33	0,95
		Alteración grave	1,52	0,63-3,68	0,35	0,68	0,18-2,56	0,57
	Perturbaciones**	Sin problemas (referencia)	---	---	---	---	---	---
		Alteración leve	1,78	0,86-3,66	0,12	1,07	0,45-2,53	0,88
		Alteración moderada	2,83	1,25-6,40	0,01	1,84	0,63-5,33	0,26
		Alteración grave	11,44	2,01-65-16	<0,01	5,66	0,41-77,31	0,19
	Síntomas depresivos	Ausencia (referencia)	---	---	---	---	---	---
		Presencia	0,73	0,47-1,13	0,16	0,78	0,45-1,34	0,37
	Diabetes mellitus	No (referencia)	---	---	---	---	---	---
		Sí	6,77	3,37-13,60	< 0,001	5,27	2,27-12,26	<0,01
	Índice de masa corporal	Normal (referencia)	---	---	---	---	---	---
		Bajo peso	1,66	0,36-7,65	0,52	2,28	0,46-11,28	0,31
		Sobrepeso	2,47	1,46-4,15	0,001	1,36	0,64-2,92	0,43
		Obesidad	4,9	2,81 -8,56	<0,001	2,81	1,11-7,13	0,03
	Índice cintura-talla	Sin obesidad abdominal (referencia)	---	---	---	---	---	---
		Obesidad abdominal	3,54	2,28-5,49	<0,001	1,7	0,79-3,68	0,18
	Índice cintura-cadera	Sin obesidad abdominal (referencia)	---	---	---	---	---	---
		Obesidad abdominal	3,11	1,86-5,18	<0,001	_LÜL	0,60-2,16	0,69

* Clasificación de la calidad del sueño mediante puntaje total de la escala de Pittsburgh

** De los subítems que hacen parte de la escala de Pittsburgh, se incluyeron en el modelo multivariado la latencia del sueño y las perturbaciones del sueño, las cuales mostraron un valor de p < 0,20 en el análisis bivariado.

^+^ Modelo de regresión logística ajustado por las variables que mostraron un valor de p < 0,20 en el análisis bivariado

Al analizar las dos principales variables independientes de interés, no se logró demostrar una asociación estadísticamente significativa de la duración o las alteraciones en la calidad del sueño medida por la escala PSQI, con la incidencia de hipertensión arterial. Sin embargo, las categorías “problema del sueño que merece atención médica” (OR = 1,6) (IC_95%_: 0,49-5,23) y “problema del sueño que merece atención médica y tratamiento” (OR =1,57) (IC_95%_: 0,3-6,85), muestran una tendencia hacia un mayor riesgo de desarrollar hipertensión arterial.

Durante el modelado, se recodificó la variable calidad del sueño para comparar aquellos participantes que tenían alguna alteración en la calidad del sueño con aquellos sin problemas del sueño, y no se encontró ninguna asociación significativa con la hipertensión arterial. De igual forma, se recodificó la variable horas de sueño para comparar aquellos participantes que tenían ≤ 5 horas y ≥ 9 horas, con aquellos que tenían una duración del sueño de 6 a 8 horas, sin encontrar asociación significativa con la hipertensión arterial.

El modelo final se ajustó de acuerdo con el inverso de la probabilidad de participación calculada en el análisis de pérdidas, encontrándose que la variable edad perdía su significancia estadística; las demás variables que habían mostrado significancia seguían manteniendo su asociación significativa con la incidencia de la enfermedad.

## Discusión

Los metaanálisis publicados llegan a la conclusión de que la mala calidad y la poca duración del sueño se asocian significativamente con un mayor riesgo de desarrollar hipertensión arterial ^13^^,^^14^. Sin embargo, se ha evidenciado que, en la población latinoamericana, solo existen dos estudios que evalúan esta asociación: un estudio de cohorte llevado a cabo en Colombia ^18^ y un estudio de corte transversal llevado a cabo en Brasil ^19^. Además, estos metaanálisis han mostrado una gran heterogeneidad (12: 87 % para calidad del sueño e 12: 79 % para duración del sueño) ^13^^,^^14^. Lo anterior muestra la importancia de realizar nuevos estudios en población latinoamericana, con el fin de determinar si estos resultados pueden generalizarse a nuestra población.

En el presente estudio, fue llamativo observar una alteración en la calidad del sueño en el 92,8 % de la población, lo cual contrasta con lo encontrado por Vargas *et al*. en población bumanguesa, en cuyo estudio solo el 26,1 % de los participantes calificaron su sueño como regular, malo o pésimo ^18^. En el estudio de Ruiz *et al*. en población colombiana, se encontró una prevalencia del 45,3 % de problemas del sueño que requieren atención médica ^12^. Por lo tanto, nuestra cohorte parece ser la primera en Colombia en mostrar una prevalencia tan grande de mala calidad del sueño.

Esto podría estar en relación con la gran prevalencia de síntomas depresivos (34,59 %), pues como se conoce ampliamente, la depresión altera de forma importante los patrones del sueño y puede, en el 80 % de los casos, generar insomnio y mala calidad del sueño ^20^^,^^21^. Además, la depresión también puede generar somnolencia diurna excesiva, especialmente si se trata de una depresión moderada a grave; esta somnolencia diurna excesiva, en primer lugar es un signo cardinal de sueño alterado o inadecuado ^21^ y, en segundo lugar, es un predictor importante de síndrome de apnea o hipopnea obstructiva del sueño ^22^.

La mayoría de la población en nuestro estudio (73,81 %) dormía entre 6 y 8 horas al día, lo cual podría considerarse como una duración adecuada del sueño según algunas organizaciones como la National Sleep Foundation ^23^. Sin embargo, nuestro estudio mostró una mayor prevalencia de poca duración del sueño que la encontrada por Vargas *et al*. en población bumanguesa ^18^. Otros estudios en población colombiana, como el de Ruiz *et al.*^12^, muestran una menor prevalencia de duración corta del sueño (2,71 %), una prevalencia similar de duración adecuada del sueño (76,14 %) y una mayor prevalencia de duración prolongada del sueño (21,15 %). Estas diferencias, probablemente, se deben a diferentes formas de categorizar las variables.

La incidencia de hipertensión arterial en nuestro estudio fue más baja que la reportada por otros estudios en Colombia, como el de Vargas *et al*., quienes reportaron una incidencia del 15,1 % en población bumanguesa ^18^. Esta menor incidencia pudiera estar en relación con un menor tiempo de seguimiento y con un mayor porcentaje de mujeres en nuestra cohorte (68,7 %), pues se sabe que los hombres presentan una mayor prevalencia de hipertensión arterial que las mujeres hasta los 45 años ^24^.

Al clasificar a los participantes de nuestra cohorte según su calidad del sueño, los resultados sugieren que, entre menor sea la calidad del sueño, mayor es la incidencia de hipertensión arterial, y esta relación podría tener su explicación fisiopatológica en una mayor activación del eje hipotálamo-hipófisis-suprarrenal y un aumento del tono simpático en pacientes con mala calidad del sueño ^25^.

Llamativamente, en nuestra cohorte había un porcentaje muy pequeño de participantes con un problema grave del sueño en la línea de base y, en esta categoría, casi la mitad de los participantes se perdieron durante el seguimiento, lo cual podría explicar por qué la incidencia de hipertensión arterial fue menor en esta categoría.

En el análisis multivariado no se encontró una asociación significativa entre la duración o la mala calidad del sueño y la incidencia de hipertensión arterial sistémica. Sin embargo, se observó una tendencia a ser mayor el riesgo en cuanto menor fuera la calidad del sueño. Si bien no se encontró una significancia estadística, el tamaño de la muestra en el presente estudio es pequeño, en comparación con el de otros en los cuales sí se ha encontrado ^16^; además, el intervalo de confianza es amplio, lo que podría sugerir la necesidad de una muestra de mayor tamaño para lograr encontrar significancia. De igual forma, en el caso de la categoría “problema grave del sueño”, pudo haber disminuido el poder estadístico para encontrar asociación debido al mínimo tamaño de la muestra en esta categoría (ocho participantes).

Por lo tanto, serían necesarios nuevos estudios con poblaciones más específicas que tengan problemas graves del sueño, para determinar si estas poblaciones tienen mayor riesgo de hipertensión arterial. Liu *et al*., por ejemplo, lograron encontrar una asociación de los mayores puntajes globales de la escala PSQI con hipertensión arterial sistémica, en una población de 9.404 adultos ^16^.

A pesar de que no se encontró un mayor riesgo de desarrollar hipertensión arterial sistémica entre los participantes que duermen cinco horas o menos u ocho horas o más, en comparación con los que duermen siete horas, el dormir seis horas se muestra como un factor protector. Este resultado debe interpretarse con cautela, pues algunas sociedades, como la National Sleep Foundation, llegan a considerar el dormir 6 horas dentro de la categoría de un sueño adecuado ^23^, razón por la cual podría mostrarse como factor protector sin que pueda clasificarse como un sueño corto. Al igual que ocurre con la calidad del sueño, nuestro tamaño de muestra es pequeño comparado con el de otros autores que lograron encontrar esta asociación, como es el caso de Grandner *et al*., con más de 700.000 adultos estudiados ^26^.

Nuestros resultados concuerdan con lo encontrado por Vargas *et al*. en población colombiana, quienes tampoco lograron demostrar asociación entre la mala calidad y la duración del sueño, y la hipertensión arterial ^18^. De igual forma, otros estudios como los de Sforza *et al*. en población francesa ^27^, y los de Thomas *et al*. ^28^ y Bansil *et al*. ^29^ en población norteamericana, muestran que la calidad y la duración del sueño no se asociaron con un mayor riesgo de hipertensión arterial. Sin embargo, estos resultados contrastan con lo encontrado por los metaanálisis en población asiática ^13^^,^^14^, y con otros estudios en población norteamericana ^30^, italiana ^31^ y española ^32^. En Latinoamérica, en un estudio de corte transversal llevado a cabo en Brasil, Quadra *et al*. evidenciaron una mayor prevalencia de hipertensión arterial en la población con peor calidad subjetiva del sueño ^19^.

Lo anterior pone en evidencia la necesidad de una mayor cantidad de estudios, con poblaciones más grandes, metodologías más homogéneas y mayor número de participantes pertenecientes a poblaciones latinoamericanas, norteamericanas y europeas, con el fin de determinar si realmente existe asociación de la hipertensión arterial con alteraciones de la duración y la calidad del sueño.

Este estudio correspondería al tercero publicado, en el que se investiga si existe asociación de la duración y la calidad del sueño con la hipertensión arterial sistémica en población latinoamericana. No obstante, es el primero entre ellos en utilizar el índice de calidad del sueño de Pittsburgh (PSQI) para evaluar esta asociación; este es un índice validado en población colombiana y que permite una estimación más objetiva de la calidad del sueño, lo que aumenta la confiabilidad de los resultados. Además, es el segundo estudio de cohorte prospectiva en Latinoamérica, después de la cohorte de Vargas *et al*. en investigar esta asociación ^18^.

La principal limitación del presente estudio, son las pérdidas durante el seguimiento; sin embargo, al analizarlas se demostró que no fueron diferenciales, por lo cual se concluyó que no afectaron significativamente los resultados finales. Además, no se contó con el monitoreo ambulatorio de presión arterial ni con la polisomnografía para el registro de las alteraciones del sueño, estudios que hubieran permitido una medición más objetiva de las variables estudiadas aquí y descartar otras variables de confusión, como el síndrome de apnea, el cual ha demostrado asociarse con la hipertensión arterial. Sin embargo, estos estudios son muy costosos y poco prácticos para grandes investigaciones de base poblacional.

En la presente cohorte, las mediciones estandarizadas han demostrado ser la mejor forma de medir estas variables en grandes estudios poblacionales ^13^^,^^18^. Además, no fue posible establecer el riesgo de hipopnea obstructiva del sueño con escalas como la de somnolencia de Epworth, ya que esta no se incluyó en las mediciones en la cohorte original.

Vale la pena resaltar que Waldman *et al*. encontraron que los pacientes pueden no ser conscientes de que sus síntomas podrían indicar un síndrome de apnea que requiere evaluación y tratamiento. Incluso después del diagnóstico, la excesiva somnolencia diurna asociada con este síndrome puede seguir afectando sustancialmente la calidad de vida relacionada con la salud y el funcionamiento diario. Esto plantearía la importancia de utilizar escalas como la de Epworth o practicar polisomnografía en futuras cohortes, para superar esta limitación ^33^.

En conclusión, las alteraciones en la calidad y la duración del sueño no se asociaron significativamente con un mayor riesgo de desarrollar hipertensión arterial sistémica en población colombiana. Estos resultados podrían ser útiles en futuros metaanálisis para dilucidar si en la población latinoamericana existe esta asociación, como sí existe en países orientales, con el fin de desarrollar políticas públicas orientadas hacia la promoción de la salud y la prevención de la hipertensión arterial.

Además, la importante prevalencia de mala calidad del sueño y de síntomas depresivos, sugiere que se necesitan políticas públicas para la promoción de mejores hábitos de sueño, además de la intervención oportuna de problemas del sueño y de trastornos del ánimo en nuestra población. Se requieren más estudios en población colombiana y latinoamericana para llegar a conclusiones definitivas.

## Archivos suplementarios

**Cuadro suplementario 1.** Descripción de las variables

	Definición conceptual	Definición operacional	Tipo
Variable dependiente			
Hipertensión arterial sistémica	Tensión ejercida por la sangre que es expulsada por el corazón contra las paredes de las arterias; cuando esa presión es continuamente alta se denomina hipertensión arterial.	Promedio de dos mediciones de la hipertensión arterial utilizando el tensiómetro digital elite, OMRON HEM-7320. Se definió tensión arterial como PAS ≥ 140 mm Hg o PAD ≥ 90 mm Hg o aquellos que estuvieran tomando antihipertensivos. Los pacientes se dividen en aquellos que tienen hipertensión arterial y aquellos que no.	Cualitativa nominal
Variables independientes			
Calidad del sueño	Hace referencia a dormir bien durante la noche, así como tener un buen funcionamiento diurno. Se evalúa mediante la escala PSQI (Pittsburgh Sleep Quality Index)	Puntaje total de la escala PSQI el cual oscila entre 0 y 21 puntos. Variable categórica: Sin problemas del sueño (≤ 4) Merece atención médica (5 a 7) Merece atención médica y tratamiento (8 a 14) Problema grave del sueño (15 a 21)	Cuantitativa discreta Cualitativa categórica politómica
Duración del sueño	Número de horas que el paciente duerme cada noche, no incluye el tiempo que permanece en cama despierto. Esta variable corresponde a la variable independiente categorizada de acuerdo con los metaanálisis de Hui Li *et al.*	Reporte del paciente con respecto al número de horas que cree que duerme cada noche. ≤ 5 6 7 8 ≥ 9	Cuantitativa discreta
Variables sociodemográficas			
Edad	Tiempo transcurrido en años a partir del nacimiento del individuo	Años cumplidos según la cedula de ciudadanía hasta el momento de la entrevista	Cuantitativa discreta
Sexo	Condición orgánica que distingue al hombre de la mujer.	Condición biológica al nacer (hombre o mujer)	Cuantitativa nominal
Estrato socioeconómico	Grupos en que se divide la población de acuerdo con el distinto poder adquisitivo y nivel social.	Categoría del estrato socioeconómico en el que se ubica la persona, según el último recibo de la luz.	Cualitativa ordinal
Raza	Grupos étnicos en que se suele dividir la especie humana, según ciertas características físicas distintivas, que se transmiten por herencia de generación en generación.	Identificación que hace el entrevistado de su raza (blanca, negra, mestiza, no sabe, otros)	Cualitativa nominal
Estado civil	Situación de las personas determinada por sus relaciones de familia, provenientes del matrimonio o parentesco, que establece ciertos derechos y deberes.	Identificación que hace el entrevistado de su estado civil. (soltero, casado, viudo, divorciado, unión libre, separado).	Cualitativa nominal
Escolaridad	Clasificación de estudio de acuerdo con los años de educación formal aprobados.	Identificación que hace el entrevistado del nivel educativo alcanzado. (Ninguno, primaria, secundaria, técnico, universitario).	Cualitativa ordinal
Ocupación	Es el oficio o profesión en el cual se desempeña el participante la mayor parte del tiempo.	Identificación que hace el entrevistado de la ocupación principal en el momento de la entrevista.	Cualitativa nominal politómica
Aseguramiento en salud	Tipo de aseguramiento social	Identificación que hace el entrevistado del tipo de aseguramiento social al que pertenece (subsidiado, contributivo, ninguno)	Cualitativa nominal
Variables modificadoras de efecto o potenciales factores de confusión			
Diabetes	Enfermedad crónica que se desencadena cuando el organismo pierde su capacidad de producir suficiente insulina o de utilizarla con eficacia.	Diagnóstico de diabetes, uso de medicamentos hipoglucemiantes o glucemia en sangre en ayunas mayor o igual a 126 mg/dl Datos obtenidos del participante y resultados del examen de laboratorio. Reportado como “diabético” o “no diabético”	Cualitativa nominal
Tabaquismo	Consumo de tabaco	Autorreporte que hace el entrevistado frente al consumo actual o previo de tabaco al momento de la entrevista. Fumador: persona que durante su vida ha fumado más de 100 cigarrillos y ha fumado, por lo menos, 1 cigarrillo en los últimos 6 meses. Exfumador: persona que habiendo sido fumador, se ha mantenido en abstinencia, al menos, por los últimos 6 meses. No fumador: aquella persona que ha fumado menos de 100 cigarrillos en toda su vida	Cualitativa nominal
Alcohol	Consumo actual de alcohol	Frecuencia del consumo de bebidas alcohólicas, reportado por el participante. No consumidor: si en el último año no había consumido ninguna bebida con contenido alcohólico. Consumidor: si había consumido en el último año bebidas alcohólicas independientemente del número de tragos consumidos.	Cualitativa nominal
Síntomas depresivos	Presencia de síntomas relacionados con depresión	Autorreporte sobre síntomas depresivos en la última semana, de acuerdo con el cuestionario CES-D (sin síntomas depresivos	Cualitativa nominal.
Índice de masa corporal	Medición de relación entre peso y talla	Cálculo obtenido de la división del peso en kg entre la estatura en metros al cuadrado. Clasificado de la siguiente manera: < 18,5: bajo peso 18,5 a 24,99: normal 25,0 a 29,99: sobrepeso ≥ 30,0: obesidad	Cualitativa categórica politómica
Índice cintura-talla	Medición de la relación de la cintura y la talla	Cociente de la cintura (cm) sobre la estatura (cm) Se define “obesidad abdominal” cuando se presenta un índice cintura-talla mayor de 0,5 tanto en hombres como en mujeres.	Cuantitativa continua
Índice cintura-cadera	Se obtiene midiendo el perímetro de la cintura a la altura de la última costilla flotante y el perímetro máximo de la cadera a nivel de los glúteos.	Cociente entre la circunferencia de cintura y la circunferencia de cadera. Definiéndose obesidad abdominal cuando se presenta un índice cintura-cadera mayor de 0,90 en hombres y de 0,85 en mujeres.	Cuantitativa continua
Actividad física	Todo movimiento del cuerpo que hace trabajar a los músculos y requiere más energía que estar en reposo.	Intensidad de la actividad física según los equivalentes metabólicos (METs) - minuto. Dato obtenido de lo reportado por el participante en el cuestionario IPAQ corto, categorizado por niveles: Leve: menor o igual a 600 METs-min Moderada: > 600 y 1.500 METs-min	Cualitativa ordinal

PAS: presión arterial sistólica; PAD: presión arterial diastólica; METs-min: equivalentes metabólicos por minuto
